# Cereal and Confectionary Packaging: Background, Application and Shelf-Life Extension

**DOI:** 10.3390/foods11050697

**Published:** 2022-02-26

**Authors:** Anna-Sophia Bauer, Kärt Leppik, Kata Galić, Ioannis Anestopoulos, Mihalis I. Panayiotidis, Sofia Agriopoulou, Maria Milousi, Ilke Uysal-Unalan, Theodoros Varzakas, Victoria Krauter

**Affiliations:** 1 Packaging and Resource Management, Department Applied Life Sciences, FH Campus Wien, 1030 Vienna, Austria; anna-sophia.bauer@fh-campuswien.ac.at; 2 Center of Food and Fermentation Technologies, Akadeemia tee 15a, 12618 Tallinn, Estonia; kart@tftak.eu; 3 Department of Chemistry and Biotechnology, School of Science, Tallinn University of Technology, Ehitajate tee 5, 19086 Tallinn, Estonia; 4 Faculty of Food Technology and Biotechnology, University of Zagreb, HR10000 Zagreb, Croatia; kata.galic@pbf.unizg.hr; 5 Department of Cancer Genetics, Therapeutics & Ultrastructural Pathology, The Cyprus Institute of Neurology & Genetics, AyiosDometios, Nicosia 2371, Cyprus; ioannisa@cing.ac.cy (I.A.); mihalisp@cing.ac.cy (M.I.P.); 6 The Cyprus School of Molecular Medicine, AyiosDometios, Nicosia 2371, Cyprus; 7 Department of Food Science and Technology, University of the Peloponnese, Antikalamos, 24100 Kalamata, Greece; s.agriopoulou@uop.gr; 8 Department of Chemical Engineering, University of Western Macedonia, 50100 Kozani, Greece; mmilousi@uowm.gr; 9 Department of Food Science, Aarhus University, Agro Food Park 48, 8200 Aarhus, Denmark; iuu@food.au.dk; 10 CiFOOD—Center for Innovative Food Research, Aarhus University, Agro Food Park 48, 8200 Aarhus, Denmark

**Keywords:** food packaging, cereals, confectionary, bakery, food quality, shelf-life, sustainable packaging, active and intelligent packaging, modified atmosphere packaging, vacuum packaging

## Abstract

In both public and private sectors, one can notice a strong interest in the topic of sustainable food and packaging. For a long time, the spotlight for optimization was placed on well-known examples of high environmental impacts, whether regarding indirect resource use (e.g., meat, dairy) or problems in waste management. Staple and hedonistic foods such as cereals and confectionary have gained less attention. However, these products and their packaging solutions are likewise of worldwide ecologic and economic relevance, accounting for high resource input, production amounts, as well as food losses and waste. This review provides a profound elaboration of the status quo in cereal and confectionary packaging, essential for practitioners to improve sustainability in the sector. Here, we present packaging functions and properties along with related product characteristics and decay mechanisms in the subcategories of cereals and cereal products, confectionary and bakery wares alongside ready-to-eat savories and snacks. Moreover, we offer an overview to formerly and recently used packaging concepts as well as established and modern shelf-life extending technologies, expanding upon our knowledge to thoroughly understand the packaging’s purpose; we conclude that a comparison of the environmental burden share between product and packaging is necessary to properly derive the need for action(s), such as packaging redesign.

## 1. Introduction

Over the past decades, global awareness about environmental, social and economic sustainability challenges, as well as the need for immediate action to limit their negative short- and long-term impacts, has risen considerably. With regard to environmental sustainability, challenges encompass, but are not limited to, the use of resources, land, water, energy, and generation of associated emissions and waste. In order to facilitate the transition towards a sustainable future, several (inter)national goals, commitments, and legal bases have already been initiated or applied. These include, for instance, the Paris Agreement on climate change and the United Nations Sustainable Development Goals (SDGs) on a global scale, the European Green Deal including the New Circular Economy Action Plan, as well as the Farm to Fork Strategy on European level and numerous implementations into national law systems [1,2,3,4,5,6].

Regarding food, it is well-agreed in the scientific community and beyond, that a great share of negative environmental impacts such as global anthropogenic greenhouse gas emissions or waste originate from food systems [7,8,9]. These systems are defined as the whole of actors and activities involved, from production to the disposal of food products of different origins, as well as herewith associated natural, social, and economic environments [10]. Moreover, they are composed of subsystems (e.g., farming) and connected to other systems (e.g., energy). A complex network in which changes (e.g., policies) made in one sector may also affect others. Against this background, different international efforts have been taken to achieve sustainable food systems, which will provide present and future generations with a secure supply of safe food [11].

Packaging is strongly associated with food, allowing, amongst other functions, containment, protection, and transportation of contents, and thus can be seen as an integral part of food systems [12,13]. Nevertheless, nowadays it is the subject of intense debates and even stricter legal requirements, mainly due to massive circularity gaps including, for example, unsatisfactory end-of-life scenarios such as limited recyclability or (marine) litter [14,15]. However, the simple omission of packaging is hardly possible, since a well-chosen packaging system frequently shows positive (indirect) effects on the total environmental sustainability of a food system by, for example, reducing food losses and food waste or increasing transport efficiency [16]. Therefore, when aiming at developing sustainable packaging solutions, it is important to apply a holistic and interdisciplinary approach over the whole life cycle of both food and its corresponding packaging [17].

Since packaging offers a service to the food product and does not fulfil an end in itself, it is often worth starting a packaging development or a redesign process from the food perspective. By gaining profound knowledge of the food product itself, together with the intrinsic and extrinsic factors that affect quality along the food supply chain, further packaging requirements can be defined and considered in the innovation process [12,13,17].

Due to their high environmental impact, the focus of research and development activities is often on (animal protein-rich) foods such as meat or milk [18,19,20]. Despite their high nutritional value that shouldn’t be underestimated, cereal and confectionary products are rather underrepresented, regarding their impact in health but also in economic and environmental sustainability [21,22,23,24,25,26,27]. For instance, about 50% of daily required carbohydrates are consumed through bread in industrialized countries. Further, cereals are also an important source of proteins, minerals, and trace elements [28]. Expressed in figures, retail sales of bread alone were expected to reach about 92 billion euros in Europe in 2021 [29]. On the other hand, confectionary products reached a production volume of 14.7 million tons with an annual turnover of 60 billion euros along with an export value of 9.2 euros and an import value of two billion euros in Europe (EU28) in 2019 [30].

In more detail, the present review aims at building a comprehensive basis for future sustainable packaging development activities in the area of cereal and confectionary products by:

- Presenting relevant information on packaging functions and properties of packaging materials,
- detailing product group specific decay mechanisms and frequently used packaging solutions,
- and highlighting packaging-related shelf-life extension technologies.

**Figure 1:**
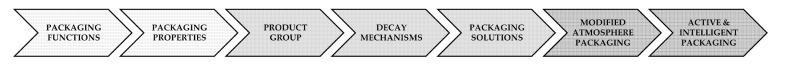
Outline of discussed topics, based on the review’s aims.

The text is therefore structured as follows: After the introduction, a general background on food packaging is discussed, followed by product group specific decay mechanisms and packaging solutions. Finally, packaging measures that can extend the shelf-life are presented (see also Figure 1).

## 2. Packaging

### 2.1. Packaging Functions

No matter how diverse individual products and packaging solutions may be on the market, it is well-agreed in relevant literature that the main functions of packaging can be broken down into a few. Next to the concept of primary and secondary functions, where the former describes in particular the technical functions like storage and transport, and the latter describes functions related to e.g., communication, a more holistic concept is frequently mentioned in the packaging literature. This concept describes the four basic functions of food packaging as (i) containment, (ii) protection, (iii) convenience, and (iv) communication [12,13,31,32,33].

Although the containment function is often overlooked, it can be considered one of the most essential, since it prevents product loss and contamination and facilitates storage, transportation, and distribution. There are only a few exceptions, where containment and thus packaging is not needed. Such examples are relatively large, chunky products that are often regionally produced and consumed within a short period of time or that show long shelf-life [12,13,31].

The protection function is often recognised as well as highlighted and can be indeed considered as the most important aspect of packaging. It limits or excludes intrinsic as well as extrinsic physical, chemical, and biological factors that may have negative influences on the quality of the respective food product. In the best case, the packaging is even capable of extending the shelf-life of the product. Therefore, it is of upmost importance to match the food product’s properties and requirements along the supply chain with packaging to achieve optimal results. Both under- and over-packaging should be avoided since this may result, on one hand, in food losses or waste and, on the other hand, in excessive packaging [12,13,31].

Further, the convenience function refers to the practical aspects or user-friendliness of packaging. As an example, easy-to-open or -empty, microwave- or heat-able, resealable, or portion packaging can be named. These features are more and more implemented in package designs, since they allow to specifically address target groups (e.g., children, elderly, single-households, on-the-go lifestyle) and therefore frequently influence the market success of a product [12,13,31].

Last but not least, the communication function allows for information transfer and marketing. While the former allows to display legally required (e.g., product name, ingredients, shelf-life), necessary (e.g., barcodes), or voluntary (e.g., certificates, cooking recipe) information, the latter enables to transfer an often unique brand image (e.g., form, colour, shape), which may be of great recognition value [12,13,31].

It is worth mentioning that a successful package on the market does not only need a strong product in terms of quality but also an effective packaging, which in a clever way combines the above described four functions of containment, protection, convenience and communication. Otherwise, it may result in a short-term success (weak product and effective packaging), a situation where the potential is not achieved (strong product and ineffective packaging), or even failure (weak product and ineffective packaging) [31].

### 2.2. Packaging Properties

From a technical point of view, the functions containment and protection are closely linked to the right selection of packaging materials which consequently poses a key decision in the development process. The available material classes cover mainly glass, metal, paper/board, (bio)plastic, as well as composite materials (laminated, coextruded, blended). Composites can consist of two or more components combined to form, for example, multilayer materials (e.g., plastic-coated cardboard) which frequently show superior functional properties (e.g., barrier) and reduced weight [31], but on the downside also reduced recyclability [34,35]. Touching upon the topic of recyclability, many packaging solutions face obstacles, if it is at the stage of collection, sorting, or in general limited technical recyclability. Not even the use of mono-materials guarantees actual recycling, as it is the case for PET trays versus PET bottles (bottles are highly likely to be recycled). On the other hand, specific combinations of compatible materials, even high barrier films, for example, metallized polyolefins, might be considered recyclable in the appropriate infrastructure [36,37]. Summing up, it can be stated that each of the named materials show advantages and disadvantages (see Table 1) and the decision for a specific material must be based on the prevailing requirements (e.g., product, supply chain, use, end-of-life). Support is often provided by material specifications and declaration of compliance documents. However, it is recommended to test the materials in question under respective conditions by means of a field or laboratory test. This ensures that deviations from the target value can be recognized at an early stage in the development process [12,13,31,38,39].

**Figure 2:**
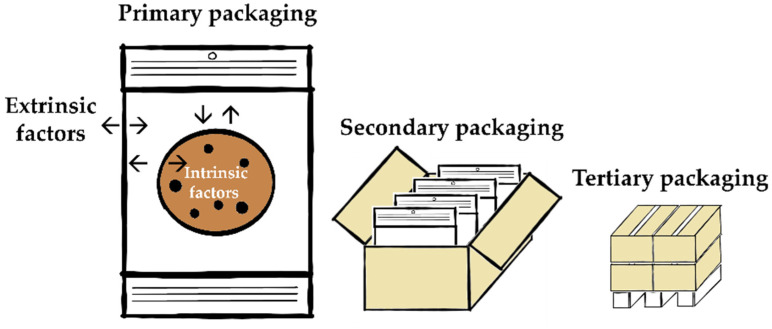
Schematic packaging levels of fine bakery ware (example: chocolate chip cookie), adapted from [12,13,31].

The key properties of packaging materials of interest are physical and mechanical strength, barrier, migration, as well as hygiene. Regarding the physical and mechanical strength, it can be noted that static as well as dynamic stress challenges the packages along the supply chain from packing, storage, and transport to consumer use. Examples for static stress are stacking and increased pressure (vacuum or modified atmosphere packaging—MAP), as well as pointed or angular products. Dynamic stress on the other hand may be caused by the production process (e.g., printing, forming, filling) or transport (e.g., vibration). The right selection of the material, but also the shape of the packaging, therefore plays a vital role in the success of a primary, secondary or tertiary package (see also Figure 2) [12,13,38,43].

Another key characteristic of materials to be considered is the barrier property. Especially, the barriers against oxygen (O_2_) and water vapour (H_2_O) transmission are determinant since these can exhibit significant influences on product quality and safety. The former for example can promote oxidation reactions, loss of quality-determining ingredients (e.g., vitamins), and growth of spoilage and pathogenic microorganisms. The latter can influence structural changes such as hardening, agglomeration, or softening of products and promote microbial growth (see Section 3.2). Additionally, barriers against carbon dioxide (CO_2_) and nitrogen (N_2_), which are the often-used gases in MAP, as well as aroma components, are decisive. Depending on the use case and product requirement, material with an appropriate barrier, i.e., permeation characteristics, should be chosen. Complementary to the above described, the barrier against other substances like fat may be considered [12,13,38,44]. Furthermore, electromagnetic radiation (light) has to be taken into consideration, since oxidative or other chemical reactions as well as structural changes may be induced or accelerated, thus impairing product quality [12,41,45,46,47].

What is important regarding chemical safety is the migration of compounds from packaging materials into the food. Migration describes the mass transfer of substances from a packaging material into the food product or vice versa. As for the permeation, the driving force behind this phenomenon is the concentration gradient. Additionally, factors such as material, storage temperature, relative humidity, and time play an influencing role [38,39,48].

Against common perception, possible migration of, for example, additives, are not only present in plastic packaging materials. Migration can also be found in other (primary or secondary (recycled)) materials such as glass (e.g., silicates), metal (e.g., corrosion of the metal, additive migration from organic coatings), paper and board (e.g., fillers, contaminations like mineral oils) and may, next to the packaging material itself, find its origin in packaging aids (e.g., labels, closures, coatings) or even set-off processes (e.g., printed and role-to-role processed or stapled materials) [12,13,38]. To ensure safety of food contact materials (including packaging), several legal requirements are in place in the European Union and beyond [39,48,49,50,51,52,53]. It should be noted that in addition to the migration from the packaging material to the food, migration processes from the food to the packaging can also be observed. This process is also called sorption or scalping and may cause alteration of the product (e.g., flavour loss) as well as reduced reusability of packaging containers due to the re-release of previously migrated substances [12,13].

In addition to chemical safety, packaging materials also play a role in the hygiene and biological safety of food products. Depending on the material used, a barrier against contamination, microorganisms and animals (e.g., food pests) can be given. To achieve a high standard of hygiene, it is crucial to utilize materials that pose a sufficient barrier and that are free from contamination. Further, it is important to use materials that do not support microbial growth. Lastly, it is important to recognise, that most packaging materials carry a low microbial count when freshly produced due to often high process temperatures (e.g., melting of glass). So, the microbial burden is often a result of recontamination during finishing processes, storage, and application, which can sometimes make it necessary to implement decontamination measures prior to the filling process [38,54].

## 3. Cereal and Confectionary Products

Against the above-summarized background, food packaging can be seen as a mediator between product and the environment, capable of significantly influencing food quality, safety, and shelf-life [12]. Regarding cereal and confectionary products, the following text aims at summarizing and categorizing the product group, presenting an overview of category specific decay mechanisms, as well as respective packaging solutions.

### 3.1. Categorization of Cereal and Confectionary Products

As shown by Belitz et al. [28], cereal and confectionary products cover a wide and diverse range of food products. They summarized different products in two groups, namely cereals and cereal products. The first group is mainly made from important staple foods such as wheat, rye, rice, barley, millet, oats and corn. These are used to produce different kinds of products. For example, Smith et al. [55] made the following division: “…unsweetened goods (bread, rolls, buns, crumpets, muffins and bagels), sweet goods (pancakes, doughnuts, waffles and cookies) and filled goods (fruit and meat pies, sausage rolls, pastries, sandwiches, cream cakes, pizza and quiche)”.

The group of confectionery products are mainly sugar-based products that, in contrast to cereal products, are predominantly consumed as a “treat” rather than a full meal. These include products such as chocolate, hard candy, and pralines [56,57]. In addition to sweet confectionery, savory snacks can also be found on the market. According to Robertson [13], these include “… a very wide range of products, including potato and corn chips, alkali-cooked corn tortilla chips, pretzels, popcorn, extruded puffed and baked/fried products, half-products, meat snacks and rice-based snacks” [13,58]. In addition to that, there are combinations of sweet and savory snacks like chocolate covered pretzels or sweet popcorn [59].

**Figure 3:**
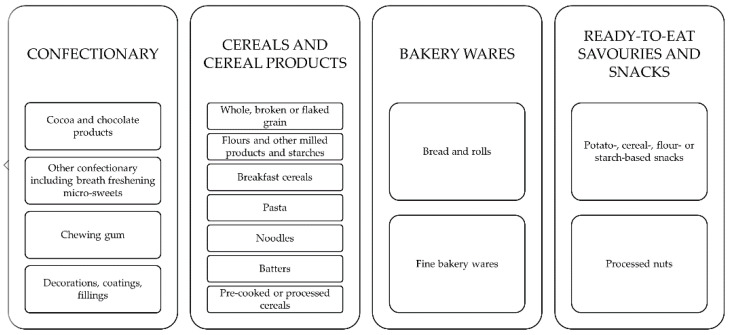
Representation of the followed product categorization. Adapted from [59].

In the available literature and other sources including statistics, codices and regulations, different approaches to properly (sub)categorize cereal and confectionary products can be found [59,60,61]. Taking a food and shelf-life perspective, it is reasonable to cluster products that exhibit similar characteristics or spoilage mechanisms. In the European Union, where there is a strong food law [62] in place, a comprehensive list can be, for example, found in the guidance document to Annex II of regulation (EC) No 1333/2008 on food additives [59,63]. For the field of cereals and confectionary, the four groups of confectionary, cereals and cereal products, bakery wares, and ready-to-eat savories and snacks are of special interest. While confectionary is further subdivided into cocoa and chocolate products, other confectionery products including breath freshening micro-sweets, chewing gum as well as decorations, coatings and fillings, cereals and cereal products are divided into whole, broken or flaked grain, flours, milled products and starches, breakfast cereals as well as pasta, noodles, batters and pre-cooked or processed cereals. For bakery wares, a classification into bread and rolls and fine bakery wares is given. Last but not least, savories and snacks are broken down into potato-, cereal-, flour- or starch-based snacks as well as processed nuts. For each of the above-mentioned subgroups, a comprehensive list of product examples is given in the mentioned document [59]. The present review adopts this categorization approach and structures relevant information on cereal and confectionary shelf-life, packaging, and shelf-life extension strategies accordingly (Figure 3).

### 3.2. Decay Mechanisms and Shelf-Life

It is well-established that intrinsic as well as extrinsic factors influence the quality of food and thus its shelf-life [13], which can be defined as the period of time a food maintains its safety and/or quality under reasonably foreseeable conditions of distribution, storage, and use [12,64,65,66]. Intrinsic factors include, amongst others, pH, water activity (a_w_), initial microbial population, redox potential value (Eh), and nutrient content and therefore determine the nature of decay mechanisms of a food product. On the other hand, extrinsic factors determine how fast decay mechanisms proceed. Typical examples are atmosphere, climatic conditions, and illumination. Packaging itself acts as mediator or separator between intrinsic and extrinsic systems [13,67]. The following paragraphs highlight the main challenges of quality maintenance of cereal and confectionary products but do not go into detail about the physical, chemical, or biological bases of these mechanisms (e.g., oxidation). This information can be found in the relevant scientific literature [13,67,68].

Focusing on cereal and confectionary products (see Table 2), moisture content (MC) and water activity (a_w_) are some of the most important quality-affecting parameters. Kong and Singh [69] define, that the a_w_ value is “…the vapour pressure of water above a sample (p) divided by that of pure water at the same temperature (p0); i.e, $a_{w}=\frac{p}{p0}$. It describes the degree to which water is free or bound to other components”. They state that this is related to “…the composition, temperature, and physical state of the compounds” [69,70]. This is of importance regarding the potential growth of microorganisms as they depend on free water presence [71].

With an a_w_ lower than 0.75, a large proportion of the products listed in Table 2 falls into the group of low-moisture or dried foods that additionally exhibit low (e.g., cornflakes) or high (e.g., crisps) fat content. In this group, water uptake and thus loss of, e.g., crispness, which occurs, e.g., in potato chips and breakfast cereals after gaining moisture at a range of 0.35 to 0.5 a_w_, is the main decay mechanism [12,13,69,80]. Other mechanisms include loss of aroma (e.g., flavoured products) or aroma uptake from the products’ surrounding due to the often porous structure of the food products. Further, structural changes such as loss of integrity due to e.g., mechanical damage (e.g., breakage), softening, or caking may occur. While microbial growth is the basis for both, low and high fat types, oxidative mechanisms, which may lead to off-odours and -tastes and subsequently to quality loss in terms of overall acceptance, are often linked to the fat content and thus tend to increase with the same [12]. Examples that can be named are nuts, chips, biscuits, and cookies. All in all, this product group can, however, be described as rather stable and therefore storage under dry and ambient conditions is recommended and possible. For example, breakfast cereals and dry pasta stay stable under temperate conditions for 6–18 months and 48 months, respectively [72,81]. Confectionary products like pulled sugar are stable for 6–9 months under temperate conditions (e.g., ~20 °C) [68].

Other products, including chocolate for example, can be allocated to compact foods with high fat content, a group mainly susceptible to the uptake of unwanted flavours and some (often minor) water exchange (uptake or loss) processes [12]. The latter can induce so-called blooming effects [13]. Sugar bloom on the one hand is often provoked by humid storage or rapid temperature changes and leads to the loss of surface gloss. Fat bloom on the other side is also known to cause quality related issues visible as a fine whitish layer [82]. Growth of microorganisms is, however, of minor importance in this product group. Storage under temperate or chilled conditions is therefore possible for up to 12–24 months [57].

Microbial growth is of major concern in the group of ready-to-eat and ready-to-cook convenience food products (e.g., fresh pasta). At this point, in addition to spoilage microorganisms, pathogenic microorganisms play an essential role [65,83]. Further, water loss and structural changes can be named. Additionally, oxidation can significantly gain importance regarding shelf-life. Accordingly, chilled storage is often preferred [13,67].

The area of bakery products can be divided into fresh bakery wares and ready-to-bake products. The first group (e.g., bread) shows high a_w_ values (>0.8) and thus short shelf-life, which is heavily influenced by water exchange processes that are often interlinked with structural changes (softening of the crust and drying of the crumb). Connected to this, starch retrogradation, which is the main mechanism of staling, can be highlighted [69]. Further, loss of moisture and hardening with a_w_ values below 0.5–0.7 [13,69,80] quickly result in low sensory acceptance of the products. While oxidation and rancidity play a minor role in this food category, uptake of flavours as well as microbial spoilage play a more elaborated role in this product group. The latter point is mainly driven by the often visible growth of moulds and yeasts on the food surface. Characteristic microorganisms are *Penicillium roqueforti*, *Hansenula anomala*, *Pichia anomala*, *Candida guilliermondii*, *C. parapsilosis*, *Saccharomyces cerevisiae*, *S. exiguus*, *S. unisporus*, *S. bayanus*, *S. pastorianus*. Additionally, *Clostridium* and *Bacillus* genera are known bacteria potentially affecting bakery wares (spore-forming), with e.g. *Bacillus* spp. causing “rope” or “ropy spoilage” (*Bacillus amyloliquefaciens*, *Bacillus subtilis*, *Bacillus pumilus*, *Bacillus cereus*) [71,84,85]. Oxidation and rancidity play a minor role in this product category. Accordingly, the average shelf-life of fresh bread and cake under ambient conditions is often less than one week [86]. In some cases, chilled or frozen storage is advisable. The group of ready-to-bake rolls show very similar decay mechanisms. However, due to the higher water content, drying and spoilage is even more pronounced. In the case of frozen products, these mechanisms are delayed. A special focus has to be laid on water exchange (freezer burn) and structural damage [87].

### 3.3. Product Group Specific Packaging

Responding to the above-mentioned predominant decay mechanisms of cereal and confectionary products, the following section aims at highlighting common packaging concepts and material choices (compare also Table 1).

Chocolate packaging has to provide a good barrier against aroma, gas (especially O_2_ and H_2_O) as well as light. This is conventionally achieved by using aluminium foil of different thickness to wrap the product. Since aluminium alone cannot be heat sealed, the per se excellent barrier of the material is, however, interrupted at, e.g., overlapping areas or gaps. Hence, diffusion (mass transfer) of aroma, gas and other molecules (e.g., mineral oil components) to the product cannot be excluded. Additionally, the originality of the product, an important factor of food safety, may not be ensured [13,67]. For this and other reasons (e.g., communication), many described packaging concepts (still) include an additional packaging layer, namely paper or paperboard [13,27,88,89,90,91,92].

Today, more and more multilayer materials can be found on the market. For example, laminates of LDPE (low density polyethylene) and aluminium allow for heat sealing of the aluminium by at the same time keeping the superior barrier and dead-fold properties of aluminium. Further, multilayer materials including paper or other aluminium replacing barrier materials (e.g., polyvinylidene dichloride (PVdC)) are available. Possible build-ups may include LDPE/aluminium/paper or LDPE/PVdC, respectively [13]. Nowadays, a shift towards packaging made (solely) from (oriented) PP, which exhibits, due to a stretching process, inter alia, improved mechanical and barrier properties, is notable [21,92]. Additionally, cold sealing, is more and more adopted, since it avoids exposing sensitive products, such as chocolate, to elevated temperatures during heat sealing. This alternative is made possible by applying cold-seal adhesives on the intended sealing areas of the packaging film and pressing of two of the sealing areas together [31].

Individually packed chocolate products, such as chocolate coated bars or pralines, are often bought for hedonistic reasons (e.g., treats, gift function) and thus the communication function (design) of these packages is frequently at the forefront [13,56]. While the functions of containment and protection are already met, these packages often use excess packaging materials and/or layers and for example consist of a (e.g., polyethylene terephthalate (PET)) tray with individual cavities, (e.g., aluminium) wrapping of the individual pieces, a (e.g., paperboard) box, (e.g., polyethylene (PE) or polypropylene (PP)) overwrapping and packaging aids (e.g., labels, stickers). Glass or metal is also used in some cases [13].

Many confections, such as hard candies, gums, toffees and caramels are likewise (twist) wrapped individually. This is either for technical reasons such as provision of an adequate (H_2_O) barrier and thus avoidance of moisture loss or uptake, resulting in e.g., drying or agglutination of the product pieces, hygienic reasons or distinction from other products. As for chocolate, tightness of the package should be in the ideal case assured [73]. Due to their in general good barrier properties and sealability, the market dominating polyolefins (PE and PP) as well as PET [93] are also frequently used in this product category (e.g., multipacks) [21,94]. If elevated barriers are needed, different multilayer materials are also adopted. Further, glass and metal packaging can be found on the market and traditional materials include waxed paper, waxed glassine and waterproof, plasticized cellulose fibre [57]. Plain paper and board are, however, hardly used as a primary packaging material, since products tend to stick to the material. The packaging types in this product category are manifold and include, for example, trays, flow packs, boxes (for example cardboard and metal) and jars [13].

Other products such as biscuits, (processed) nuts and fruits are traditionally packaged in regenerated cellulose (trade name Cellophane) fibres (RCF). Therefore, RCF is usually coated with either LDPE or PVdC copolymer and often with a layer of glassine in direct contact with the product if it contains fat. Currently, this combination of materials is replaced by PP, either as plain or pearlized OPP film, coextruded OPP (OPPcoex) film, or acrylic-coated (Ac) on both sides. Plain OPP films require a heat seal coating to improve sealability while coextruded OPP provides superior seal strength. If a high O_2_ barrier is required, then acrylic-coated OPP (AcOPP) is used. One side is sometimes coated with PVdC copolymer rather than Ac. In addition, Ac and PVdC copolymer-coated OPP films provide a superior flavour and aroma barrier compared with that of uncoated OPP. Biscuits are often packed in PP and additionally a cardboard box, acting as secondary packaging [13,25].

In comparison to other products, the dry and low in fat group of cereals and cereal products, (such as whole, broken, flaked or milled) grains (e.g., wheat and rice) show rather low packaging demands. Mostly used are paper bags, flexible plastic bags (e.g., PE [95]), as well as cardboard boxes [96,97]. There are also variations of these packages, for example inner flexible plastic bag and a secondary cardboard box. If paper is used and high barriers are needed, LDPE liners for example can be applied [13], also to avoid mineral oil migration [98]. Rigid laminates with paper content and plastic lids usually known in snack product packaging, are also available. Flours for example are commercially packaged in bags or bulk bins [13]. In addition to that, woven PP bags are commonly used in developing countries. However, Forsido et al. [99] discussed that the low moisture barrier led to chemical, physical, sensorial, and microbial changes of flour. Another successful approach for flour packaging that was used for decades, was bags made from cotton twill [13].

The barrier requirements for breakfast cereals packaging are set higher than in the above-mentioned group since crispness, formation of off-flavours, loss of aroma and vitamins or breakage are more critical for consumer acceptance [13]. Consequently, the inner packaging/primary packaging level of these products is a plastic bag, mostly HDPE (high density polyethylene), giving a sufficient water vapour barrier since moisture vapour transmission rates less than or equal to 15 g/m^2^-day-atm are often required. Sealant polymers such as EVA (ethylene vinyl acetate), ionomer, mPE (metallocene polyethylene), or blends are used for low temperature seals, form-fill-seal packaging, and easy opening seals [95]. In order to increase barrier characteristics, HDPE is also coextruded with a thin layer of EVA or PA (polyamide) and EVOH (ethylene vinyl alcohol) polymers [95,100]. Other O_2_ barrier materials for breakfast cereals are PVdC and coated polypropylene-low density polyethylene [101]. In addition, PP-bags are common liners. The secondary packaging/outer packaging is most frequently a fibreboard box [13,22]. Alternative packaging concepts include coated paperboard, plastic cups, as well as metal boxes and glass jars [13,102].

Dried pasta is often packaged in paperboard carton, containing a plastic window. At the moment, most pasta products are packaged in plastic films, such as PE or oriented polypropylene [13,103,104,105,106,107]. For fresh pasta/noodle products, packaging solutions might be different, as appropriate barriers (gas and/or water vapour) and/or MAP (e.g. CO_2_:N_2_ 20:80% MAP for pasta) is needed [107,108]. The selection of packaging materials for fresh pasta products can also depend on whether or not the product is pasteurized (thus, the package must be able to withstand the pasteurization conditions) and whether or not the product is to be heated in its package (the package must be able to withstand either heating in boiling water or microwave conditions) by the consumer. For products which are not pasteurized nor intended to be heated in their package, a rigid tray of PVC-LDPE sealed with PA-LDPE film is common. When microwave heating is used, the rigid tray is usually made from crystalline polyethylene terephthalate (PET-C), or polystyrene-ethylene vinyl alcohol copolymer-LDPE (PS-EVOH-LDPE) laminate, and the film may be based on PVdC copolymer-coated PET, OPET-EVOH-LDPE, or PP [109].

Packaging of fresh bakery products such as bread is a moisture balancing act. On one hand, moisture needs to be contained to prevent drying of the product and on the other hand, moisture has to be released from the product to avoid softening of the crust and microbial spoilage. Since there is a wide range of products and product characteristics, also a wide range of packaging solutions can be found. Frequently, paper-based materials, LDPE, LLDPE, HDPE bags as well as OPP, either as plain, pearlized, OPPcoex, or Ac/OPP/Ac films are used [13,95,110,111,112,113,114]. The bags are usually closed either with a strip of adhesive tape or a (plastic) clip in order to reduce moisture loss [111,113,115]. EVA polymers are also used for sealability and optics [95]. Perforated LDPE bags are used (for crusty products) in order to prevent the formation of a leathery consistency of the crust due to moisture migration from the crumb [115]. If aroma and taste barriers are needed, PA is used [95]. Vacuum packaging including the use of respective barrier packaging materials is only used in some exceptions (e.g., flat breads) in this product category due to mechanical impairment of the often soft products. MAP rich in CO_2_ is whereas more frequently used (e.g., sliced bread, convenience applications). For example, CO_2_:N_2_ 60:40% MAP for bread, cakes, crumpets, crepes, fruit pies and pita bread. This is also the case for ready-to-bake products, which are intended to have a longer shelf-life [13].

Packaging for fried snack foods such as potato or tortilla chips, which exhibit, due to their production process, low moisture and high fat contents, preliminarily aims at providing a barrier against gases (H_2_O and O_2_) and light to avoid loss of crispness and increased oxidation/rancidity levels of the product [95]. Hence, these products are mainly packaged in high barrier multilayer films containing aluminium foil or metallisation (e.g., PET/Alu/LDPE; PETmet/LDPE; BOPP/BOPPmet) [31,94,116]. In addition, barrier polymers such EVOH or PVDC can be found in these materials. Further, rigid multilayer paper solutions with aluminium (for example spiral wound paper-board cans) or metal cans are also used. Since extruded and puffed snack foods exhibit lower fat levels and thus primarily rely on a package that provides a barrier against water vapour; these products are less often packaged in metallized materials. An example is OPP/LDPE/OPP [95]. In both scenarios, and whether flexible or rigid packaging is adopted, modified atmosphere packaging is frequently used. For example, the package is usually flushed with an inert gas (N_2_) before closing [116]. Additional mechanical protection of the often fragile products and dry storage is recommended. This might lead to the use of secondary packaging, such as cardboard boxes [31].

## 4. Shelf-Life Extension

**Figure 4:**
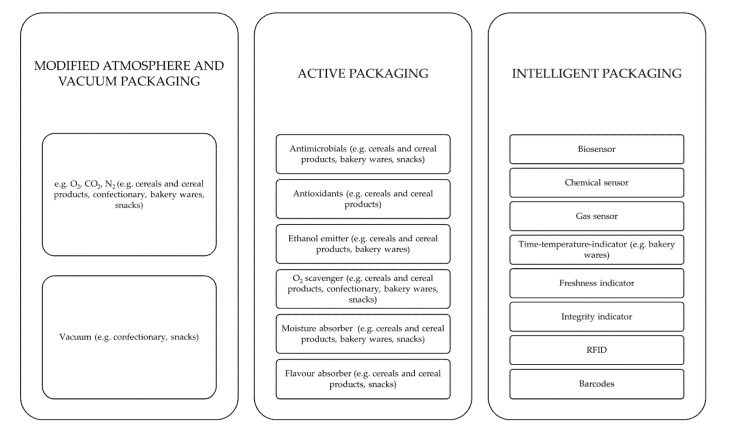
Selected examples of modified atmosphere, vacuum, as well as active and intelligent packaging approaches with certain use cases for cereal and confectionary packaging. Adapted from [13,108,117,118,119,120,121,122,123,124,125,126,127,128,129,130,131,132,133,134,135,136,137,138,139,140].

As can be seen from the above text, choosing the right packaging material concept can have a positive effect on quality maintenance and therefore shelf-life of cereal and confectionary products and food in general. Where particularly sensitive products (e.g., high a_w_ value, high fat content or oxidation potential) are present (e.g., fresh pasta, fried snacks) or an elevated shelf-life has to be achieved (e.g., ready-to-bake rolls, fine bakery wares), modern packaging concepts such as modified atmospheric packaging or active (AP) and intelligent packaging (IP) are used (combined abbreviation: AIP). Manifold different approaches can be found regarding MAP, AP, and IP, each with different relevance for the discussed product subgroups, cereals and cereal products, confectionary, bakery wares and ready-to-eat savouries and snacks. However, for an impression of these, Figure 4 depicts selected examples.

Using these approaches, other product preservation actions (e.g., heating, use of preservatives) may be reduced, which supports attempts to reach a healthier diet (e.g., reduction of salt) or a clean label (e.g., avoidance of excess additives) [141] These allow specifically addressing other remaining challenges in the chemical, biological, mechanical, and physical fields [12,13]. Thus, they are also often implemented in the hurdle technology, a concept of combining diverse adverse factors or treatments to control microbial growth in food products [13,142]. According to studies found, also biobased and/or biodegradable packaging material is experimentally combined with AIP approaches. These materials offer new opportunities, for example in making use of different barrier properties, that allow a certain shelf-life extension [134,135]. Examples for MAP and AP with traditional as well as biobased/biodegradable packaging materials can be found in Table 3.

### 4.1. Modified Atmosphere Packaging (MAP)

Leaving quality sensitive products exposed to atmospheric conditions (gas composition of N_2_, O_2_, Ar, CO_2_, traces of other gases) can trigger undesirable changes such as quality-related oxidative decay or growth of (non)pathogenic aerobic microorganisms. On the contrary, modifying the atmosphere inside a packaging can help maintain the quality of a product over an elevated timeframe. Consequently, common mitigation strategies include the reduction of packaging headspace and, thus, total available atmosphere or even removal of the atmosphere (to a value below one percent), which in turn results in vacuum packaging. To maintain these conditions over time, it is necessary to assure an appropriate containment function of the packaging by choosing packaging materials with an appropriate gas barrier and proper sealing. Challenges in this case are often the structure of the products and the corresponding residual oxygen in the packaging in the case of e.g., pores and the collapse of the product in the case of e.g., a soft structure [13,125,146].

A more advanced modification can be found in a so-called modified atmosphere packaging, MAP [147]. Here, an active modification takes place in a two-step process, where first the initial atmosphere is removed (vacuum) and then replaced with a specific artificially composed atmosphere before closure of the barrier packaging. Commonly, in product-dependent concentrations used, colourless and odourless gases in MAP mainly encompass CO_2_ and N_2_. Due to its formation of hydrated carbonate species in aqueous phase CO_2_ is valued for its bacteriostatic and fungistatic effect, which increases with increasing concentration. Due to the solubility in water and fat, formation of under-pressure in the package and, consequently, possible collapse of the latter is possible. To avoid this and to act as a filler gas, the inexpensive and inert N_2_ is applied. Hence, passively, also this gas contributes to quality maintenance of the product. Furthermore, O_2_ is a frequently used gas but of little relevance for the cereal and confectionary sector. Its field of application is mostly in meat (e.g., bright-red colour preservation via high-oxygen MAP) and fish products and to lower extent in plant products [145,148,149]. More recently, permitted noble gases such as argon are subject to research but not broadly applied on cereal and confectionary products [150,151]. Depending on the chosen MAP gas composition, food shelf-life can increase manifold (50–400%) and with this advantage along the supply chain can be recorded (e.g., less food waste, longer remaining shelf-life, less frequent production and transport). However, disadvantages linked to MAP, in general encompass the need for more sophisticated packaging materials and filling equipment, costs for gas and increased packaging volume [13].

Regarding the food categories at the centre of the present review, confectionary products are less frequently in the centre of research and application of MAP than cereals and cereal products, bakery wares or ready-to-eat savouries and snacks (see Table 3). One case of MAP use, however, is reported by Mexis et al. [119], for dark chocolate with hazelnuts. The authors found, that when conventionally used aluminium packaging together with storage under surrounding atmosphere was replaced with a PET/LDPE or PET-SiO_x_ packaging and vacuum or N_2_, the shelf-life (dark storage at 20 °C) was increased from 8 to 8–9 and 11 months, respectively. Also Kita et al. [152], investigated the effects of different packaging types and shelf-life extension strategies for chocolate coated products (fruits and nuts). They analysed air, vacuum and MAP (N_2_ ≥ 98%) of coated cherries, figs, hazelnuts and almonds in long term storage conditions in three different types of packaging. PP film closed with a clip was chosen for air, PP film sealed for vacuum and metallized sealed film for MAP. They resumed that the best packaging solutions for the chosen chocolate coated products, ensuring quality (for example bioactive compounds, antioxidative activity) were, on one hand, air and vacuum packaging for fruits, vacuum packaging for hazelnuts and MAP for almonds.

In the category of cereals and cereal products, and in more detail in fresh pasta, MAP often contains elevated amounts of CO_2_ (up to 80%) and corresponding low N_2_ values (balance) [13,108,120,121]. For instance, Lee et al. [120] conducted a comparative study on fresh pasta packaged under air (PS tray with PVC film) and under CO_2_:N_2_ 78:22% MAP (PA/EVOH/LLDPE). As a result, the shelf-life was doubled from 20 to 40 days at a storage temperature of 8 °C. Even higher rates of shelf-life increase for fresh filled pasta were shown in two other studies [108,121]. In the first case, samples included fresh pasta filled with cheese in a sealed tray (EVOH/PS/PE) with a barrier film (EVOH/OPET/PE) and two different atmospheres (air; CO_2_:N_2_ 50:50% MAP). Quality maintenance was increased from 7–10 days up to 42 days [108]. Similarly, in the second case, gluten-free fresh pasta was packaged in trays (control: PET; test: EVOH/PS/PE) sealed with films (control: PET; test: EVOH/OPET/PE). Shelf life under air was compared to CO_2_:N_2_ 30:70% MAP. Here, an increase from 14 to 42 days was notable [121].

Turning to bakery wares such as (pita)bread, cakes, crumpets, crepes, (fruit)pies, Robertson [13] reports a frequent use of CO_2_:N_2_ 60:40% MAP. However, in the scientific literature, a more diverse application of CO_2_:N_2_ MAP can be seen. For example, Rodriguez et al. [126] investigated extending the shelf-life of bread using MAP packaging in a combination with preservatives. The research referred to bread slices packaged in a 60 µm bag. The results showed that in the samples without added preservative, and CO_2_:N_2_ 50:50% MAP, the increases in shelf-life were 117% and 158% (at 22–25 °C and 15–20 °C). For the samples with calcium propionate addition and in N_2_ 100% MAP, shelf-life was increased by 116%. Furthermore, calcium propionate addition and CO_2:_N_2_ 20:80% MAP increased the shelf-life by 150% and 131% at 22–25 °C and 15–20 °C. When the CO_2_ concentration was increased to 50%, the increased shelf-life of the samples with added preservative was 167% at 22–25 °C. For the same settings at 15–20 °C the increase was even 195%. Fernandez et al. [149], conducted a similar research with soy bread. They as well used different settings of MAP and preservative adding but expanded the question of packaging options. They used two multilayer packaging solutions, high and medium barrier. The high barrier was LLDPE/PA/EVOH/PA/LLDPE, whereas the medium barrier solution was LLDPE/PA/LLDPE. As controls, LDPE and air atmospheres were used. The combination of high barrier packaging in CO_2_:N_2_ 50:50% or CO_2_:N_2_ 20:80% MAP without calcium propionate addition extended the shelf-life of the samples by at least 200%.

Turning to ready-to-eat savouries and snacks (e.g., crisps) Sanches et al. [128] investigated inter alia the effects of different packaging atmospheres under 40 °C and room temperature on multiple crisp samples, linked to lipid oxidation. They included marketed products under unknown MAP concentrations, air, N_2_, vacuum and oxygen scavengers in the analysis. Reflecting changes in the fatty acid profile of the crisps, it was resumed that changes in the package’s atmospheres, mostly cutting out oxygen, was crucial for the shelf-life of the crisps. Vacuum packaging options would also allow stable lipid profiles, however, they are not suitable for easily breakable crisps. Del Nobile [129] was similarly questioning the optimal packaging for crisps, however, focused on finding the best headspace gas composition for two different multilayer film packages (metallized PP and PVdC coated PE) through simulated storage. He proposed that N_2_ combined with water vapour would lead to a shelf-life extension up to 80%.

### 4.2. Active and Intelligtent Packaging (AIP)

While MAP is firmly established in the market, active and intelligent packaging has not yet reached its full potential in food packaging applications but is at the threshold of more widespread use in the European market and subject to intense research and development activities [153,154,155]. Accordingly, the following paragraphs aim at outlining the concept of AIP and highlighting applications most relevant for cereal and confectionary packaging.

Just as conventional packaging applications, AIP define as food contact materials as given in Regulation (EC) No 1935/2004. While conventional packaging has to be sufficiently inert not to transfer substances to the food in quantities that endanger human health or bring an unacceptable change of the food product (composition, organoleptic properties), AIP are intentionally designed not to be inert. This allows them to actively maintain or even improve the quality or shelf-life of food products [39]. Hence, AIP deliberately includes “active” components that are either aimed to be released to the food or that aim at absorbing substances from it. This justifies the division of active packaging into so-called releaser and absorber systems. However, a clear distinction is made to traditional substance releasing materials (e.g., wooden barrels) in food contact. The use of active substances aimed to be released to the food must also comply with the Directive 1333/2008 on food additives and should be authorized accordingly by applicable community provisions [63]. Furthermore, specific requirements regarding labelling and information, avoidance of misleading consumers as well as safety assessment and authorisation is given [39]. In addition to Regulation (EC) No 1935/2004, Commission Regulation (EC) No 450/2009 gives specific rules for the use of AIP (e.g., community list of allowed substances for use and evaluation of these) [39,156].

In response to major challenges in food quality and safety [12,13], key technologies in the area of active packaging are emitters (e.g., CO_2_, ethanol, antimicrobials, antioxidants) and scavengers (e.g., O_2_, CO_2_, ethylene), absorbers (e.g., H_2_O, flavour and odour), self-venting packages, microwave susceptors, and temperature control packaging [13,40,157,158,159,160,161,162,163,164,165]. Intelligent packaging on the other hand refers to packaging that monitors the food product and provides information about its condition [39]. Related key technologies are mostly indicators and sensors (e.g., time, temperature) and linked processing and communication systems (e.g., (printed) electronics). Further, tamper evident packaging and anti-counterfeiting applications exist [163,166]. 

Due to their effectiveness, the growth forecasts for AIP in the coming years are high, but it must be emphasised that the sustainability of such sophisticated packaging solutions should be evaluated case by case [167]. In addition to the actual reduction of food losses and food waste, factors such as, e.g., the recyclability of AIP, which may include metal-based components, should be evaluated [153,163,168,169].

Going into detail about cereal and confectionary packaging (see also Table 3), an application example for oxygen absorbers is in sliced bread. Where O_2_ concentration decreased below 0.1% within a few days of packaging, microbial shelf-life was shown to be extended. It was reported that there was no effect on sensory quality [170]. Oxygen absorber can also be used in combination with MAP. In 2003, Del Nobile et al. [127] showed that the application of CO_2_:N_2_ 80:20% MAP in the packaging of durum wheat bread prolonged the shelf-life from 3 to about 18 days at 30 °C. However, if the packaging film itself possesses a high barrier against oxygen, neither the use of scavengers nor MAP are necessary to achieve the desired shelf-life of white bread [171]. Finally, an oxygen scavenger system, consisting of a multilayer coextruded bag associated with an oxygen scavenger, was tested in different storage conditions (accelerated storage, room temperature, refrigerator), for its effect on preservative-free tortillas shelf life. The results indicated a protective effect of the packages including the oxygen scavenger system. Specifically, the weight and thickness of flour tortillas under room temperature conditions could be maintained, opposed to respective decreases detected in control packages (consisting of LDPE/LLDPE). In parallel, yeast and mold growth were hold back in the packages containing the oxygen scavenger versus control (room temperature and accelerated storage). Under refrigerated conditions, a shelf-life up to 31 days was estimated, however, independed of the use of oxygen scavengers [172].

It has been also shown that the use of ethanol emitters extend shelf-life even without establishment of an additional modified atmosphere. For ciabatta, a shelf-life of 16 days, at 21 °C could be obtained, packaged in air atmosphere and ethanol emitter addition [122].

Antimicrobial, antifungal, and antioxidative agents as active food packaging include multiple research topics. Options include the applications of essential oils, edible films, and nanocomposites, which are often used with products susceptible to microbiological degradation, e.g., sliced bread. For example, oregano essential oil has been observed to be a successful application against yeasts and moulds in sliced bread. It was applied in the form of antimicrobial sachet at concentrations of 5, 10, and 15% (*v*/*w*) at room temperature [136]. In addition to that, methylcellulose edible films produced with clove and oregano essential oil have displayed antimicrobial activity against spoilage fungi in bakery products and have improved sliced bread shelf-life to 15 days, at 25 ± 2 °C [137]. Also, cinnamaldehyde was used as an active ingredient to increase the shelf-life of sliced bread. It was incorporated in gliadin films (5%), which allowed to keep the quality of the product for 27 days of storage at 23 °C [173]. Next to having antimicrobial effects, essential oils are also antioxidative agents that can be included in packaging material like HDPE, LDPE, EVA. Zhu et al. [138] for example tested this approach with sesame essential oils for the packaging of oat cereals. However, there are also biological threats that could shorten the shelf-life of cereal and confectionery products. Essential oils from garlic, black pepper, ginger, fennel, and onion already have been tested as insect repellents for grain packaging. All these tested essential oils were characterized by significant fumigant insecticidal properties. For example, allyl mercaptan deriving from allium plants added as a sachet with rice flour, was proven as potential protective active packaging against *S. oryzae* contamination leaving sensory properties unaffected [174]. In general, the incorporation of essential oils in packaging materials is a growing sector [175,176]. One background can be that they are waterproof, so it could be the ideal material for the incorporation into a film, which will turn it from a conventional packaging material to an active one, increasing both its value and its functionality [175].

One further option of active packaging is the targeted use of composites at the nanoscale, whether organic (oils/proteins/carbohydrates) and/or inorganic, e.g., clays. This topic is of interest as active agents might have different properties in smaller scales. Materials of which at least one of its external dimensions belongs to the nanoscale (1 to 100 nm) are considered nanomaterials [177,178]. They are characterized for their unique properties such us high surface-area-to-volume ratio, fine particle size, and high reactivity [179]. One common area of research interest is represented by publications including essential oils. For example, bio-nano-composite films prepared with corn starch incorporated with chitosan nano-clay, and further enriched with a variety of ratios of grapefruit seed extracts have been studied. It was shown that this solution was capable of inhibiting fungal proliferation for a period of 20 days, compared to that of 6 days in bread packaged samples with synthetic plastic, indicating a successful active packaging approach to extent the shelf-life of bakery products [133]. Furthermore, two different formulations mainly consisting of essential oils from several plants were evaluated for their potential antifungal properties in maize grains. Specifically, in a recent study, bioactive EVOH films including various essential oils have been characterized. Cinnamaldehyde, citral, linalool and isoeugenol were investigated to decrease the activity of *A. steynii* and *A. tubingensis* strains. It was shown that the ochratoxin A production by these strains in partly milled maize grains could be reduced significantly. The inhibitory effect was the highest in EVOH with cinnamaldehyde, followed by isoeugenol and citral [180]. In parallel, EVOH copolymer films incorporated with essential oils from *Origanum vulgare*, *Cinnamomum zeylanicum* and/or their major active constituents have been studied. The results showed that carvacrol and cinnamaldehyde were effective in decreasing *Aspergillus flavus* and *A. parasiticus*-induced aflatoxin production in maize, respectively. Overall, cinnamaldehyde showed the highest inhibitory effect, followed by combinations of EVOH with essential oils from *Origanum vulgare*, *Cinnamomum zeylanicum* and carvacrol [181].

Next to these highly discussed organic nanoparticles, inorganic particles like Ag (silver) and TiO_2_ (titan dioxide) have also been applied to packaging solutions, for example cereal products, due to their antimicrobial effects [182,183,184,185]. However, there is a concern on potential risk of nanoparticles migrating into food, although limited data showed that obtained values were within the limits set by the legislation [185,186,187,188,189]. It was shown that Ag-TiO_2_ nanocomposite incorporated in HDPE considerably extended shelf-life and microbiological safety of bread in comparison with control sample stored in an open atmosphere or in HDPE bags [144]. Not only the characteristics of plastic packaging can be optimized by the inclusion of nanoparticles. The modification of paper with Ag-TiO_2_-SiO_2_ (silicon dioxide) or Ag/N-TiO_2_ composites can improve the papers material characteristics. It was shown that such paper was capable to extend the shelf-life of bread by 2 days in comparison to the control, in both ambient (18–20 °C) and refrigerated (0–4 °C) conditions [190].

Research in optimizing packaging with nanostructures goes even further to high-tech materials. An example is a packaging material with a montmorillonite layer. It was shown that montmorillonite composite polyamide 6 nano-fibres placed over PP films, increased the shelf-life of bread by 2 days at room temperature, due to inhibition of microbial growth [191].

Intelligent packaging, on the other hand, is a special packaging technique aiming to monitor the quality of the packaged food and to predict or measure the safe shelf-life better than a best before marking date [122,130,171,192,193,194]. It provides functions beneath the ones considered as conventional e.g., protection and containment and is used to monitor the condition and provide quality information of packed foods to the consumers [158]. Different indicators, such as time-temperature, microbial growth, product freshness, pack integrity etc., are used as intelligent packaging systems. High temperatures and/or temperature fluctuation are often correlated with food deterioration as result of detrimental biochemical reactions combined with microbial growth. Depending on the food sensitivity specific intelligent indicators can be applied to specific food products. The time-temperature indicator measures the change that imitates the targeted quality characteristics with the same behaviour under the same time-temperature exposure. The pH and enzymatic indicators can also give information about the quality of food [195]. Commercially available time-temperature indicators can be used to monitor quality changes of many perishable and semi-perishable foods. Among other products, these indicators have been applied to canned fruitcake for 10 days’ storage at constant (12, 25 and 37 °C) temperatures. Sensory analysis, as quality characteristic of the product, was correlated with indicator response [140,196].

Reflecting the above chapters and findings, it can be summarized and confirmed that, if chosen correctly, cereal and confectionary packaging, as well as food packaging in general can make a valuable contribution to maintaining the quality and safety of food [12,13,17]. Accordingly, it can also help to prevent food losses and waste, an important point when it comes to making our food systems more sustainable [11,16]. This point is also taken up in the SDGs and influences current political efforts such as the European Union's Green Deal [2,3,6].

However, packaging redesign or optimizations should not simply be carried out without evaluating the effects on ecological, social, and economic sustainability as objectively as possible. This is the only way to avoid possible hidden trade-offs [17].

In addition, close cooperation between a wide range of disciplines is required. In this context, and among others, material science, sustainability science and social sciences, and humanities can be mentioned in addition to food science and technology. The latter in particular has, however, an important enabling function [197,198]. The future focus here could be on the points of promoting (i) diverse and sustainable primary produce, (ii) new processes and systems for sustainable manufacture, (iii) reduction of food and material waste along the supply chain, (iv) safety and traceability, (v) affordable and balanced nutrition, (vi) healthy diets as well as (vii) digitalization. MAP and AIP are important approaches in this context, which are particularly present in the points (ii), (iii) and (iv) [198].

## 5. Conclusions

The ongoing discussion about packaging optimization towards the enhancement of the sustainability of certain products, asks for a profound review of the status quo in specific food groups. Cereal and confectionary were found to be underrepresented in recent publications addressing this topic, despite their global economic and ecologic importance. To take the right steps aspiring more sustainable production and consumption of goods, it is essential for practitioners along the food supply chain to thoroughly understand packaging functions (containment, protection, convenience, communication), properties (physical and mechanical strength, barrier, migration, hygiene), product group specific decay mechanisms, used packaging solutions, and shelf-life extension strategies.

Commonly available packaging solutions vary in material selection (glass, metal, plastic, paper), as well as in shape (rigid, semi-rigid, flexible) and size. Therefore, each packaging solution offers unique benefits and limitations regarding its optimization potential. Important decay mechanisms mediated by packaging in cereal and confectionary products and snacks include inter alia oxidative mechanisms and changes in moisture content. Especially for products for which quality is easily impaired through such mechanisms, packaging solutions and technologies extending the shelf-life need to be considered as ways to improve the products´ sustainability. This, in combination with a proper material selection, includes the applications of MAP and AIP (e.g., scavengers, indicators, active ingredients) as well as novel approaches (e.g., nanotechnology).

However, sustainability improvement includes different other aspects. After the proper understanding of the packaging’s purpose in these certain product categories and subcategories, the question of burden shares between the environmental impacts of the food product itself in comparison to its packaging must be considered along the whole life cycle. Thus, further research is deemed necessary to investigate data from related Life Cycle Assessment (LCA) studies and to combine the findings with the current status quo, in order to derive proper redesign steps for cereal and confectionary products. However, LCA is by default limited to environmental analysis and does not cover all sustainability dimensions. The inclusion of economic and social aspects would finally provide a holistic picture on how to attain more sustainable products.

## Figures and Tables

**Table 1:** Overview of the properties and applications of most widely used materials for packaging.

Packaging Material		Barrier			Heat Seal-Ability	Mechanical, Physical and Chemical Properties	Application	Reference
		Oxygen	Moisture	Light				
Plastic	Low-density polyethylene (LDPE)	Very low	High	Low	Yes	Toughness, flexibility, resistance to grease and chemicals, temperature range −50 – +80 °C	Bags, flexible lids and bottles	[12]
	Linear low-density polyethylene (LLDPE)		High			Toughness, extensibility, resistant to grease, temperature range −30 – +100 °C	(Strech) wrap	
	High-density polyethylene (HDPE)		Extremely high			Toughness, stiffness, resistance to grease and chemicals, easy processing and forming, temperature range −40 – +120 °C	Bottles, cardboard liners, tubs, bags	
	Polypropylene (PP)	Low	High	Low	Yes	Moderate stiffness, strong, resistant to grease and chemicals, temperature range −40 – +120 °C	Bottles, cardboard liners, tubs, microwavable packaging, bags	
	Polyethylene terephthalate (PET)	Good	Good	Low	Yes	Stiffness, strong, resistance to grease and oil, temperature range −60 – +200 °C	Bottles, jars, tubs, trays, blisters, films (bags and wrappers)	[12,40]
Glass	Transparent	Absolute		Low	No	High temperature and pressure stability, brittle, chemical resistance, microwave-able	Bottles, jars	[12,40,41,42]
	Green			Good				
	Brown			High				
Metal (aluminium, tinplate, tin-free steel)		Absolute			No	High temperature stability	Bottles, cans, tubs, caps	[12,40]
Paper and board			Extremely low	High – extremely high	No	Mechanical stability	Boxes, liners	[12,40,41]

**Table 2:** Water activity and moisture content of confectionery products, breakfast cereals, snacks, and bakery products.

Product category	Subcategory	Product	Water Activity [a_w_]	Moisture Content [%]	Reference
Confectionery	Cocoa and chocolate products	Chocolate	0.42–0.60	1.2	[72]
	Other confectionery including breath freshening micro-sweets	Hard candy	0.25–0.40	2.0–5.0	[73,74]
		Fudge, toffee	0.45–0.60	6.0–18.0	
		Nougat (white, dark)	0.55	8.00–10.0	[13,75]
		Jelly, liquorice	0.50–0.75	8.0–22.0	[73,74]
		Marshmallow	0.60–0.75	12.0–22.0	
		Marzipan	0.75–0.80	–	[13]
	Chewing gum	Chewing gum	0.40–0.65	3.0–6.0	[73,74]
Cereals and cereal products	Whole, broken, or flaked grain	Oats, grains, cereals	0.34–0.70	8.8–9.2	[13,72]
	Breakfast cereals	Cornflakes	0.25–0.38	1.7–3.5	
		Puffs	0.17–0.20	0.48–1.70	
	Fresh pasta	Fresh pasta	0.91–0.98	≥24	
	Dry pasta	Dry pasta	0.33–0.57	5.4–8.3	
Bakery wares	Fine bakery wares	Sponge cake, muffins	0.84–0.95	21.0–40.0	[76,77]
		Croissant crust	0.59–0.61	8.0–10–0	
		Croissant crumb	0.92–0.94	30.0–33.0	
		Biscuits	0.60–0.63	1.5–3.0	[72,78]
		Wafers	0.13–0.15	2.1	[72]
		Cookies	0.18–0.64	1.4–11.7	
	Bread and rolls	Flat bread (no yeast)	-	33.0–35.0	[79]
		Sourdough bread, yeast bread crumb	0.91–0.95	29.0–40.0	[72]
		Sourdough bread, yeast bread crust	0.88–0.94	26.0–32.0	
		Bagel crust	0.96	38.5	
		Bagel crumb	0.92	31.0	
Ready-to-eat savouries and snacks	Potato-, cereal-, flour- or starch-based snacks	Popcorn	0.07	0.28	
		Chips	0.09–0.27	0.3–1.3	
		Crackers, grissini, sticks, pretzels	0.05–0.54	1.1–5.4	
	Processed nuts	Nuts, seeds, nibs	0.15–0.75	0.5–3.1	

**Table 3:** Effects of packaging material selection, active packaging (AP) and modified atmosphere packaging (MAP) on shelf-life extension of cereal and confectionary products. Abbreviations: m = month; d = day; RH = relative humidity; RT = room temperature.

Category	Product	Packaging Material	AIP/MAP Applied	Storage	Shelf-Life	Reference
Confectionary	Dark chocolate with hazelnuts	Alu (commercial)	Air	20 °C in dark	8 m	[119]
		PET/LDPE	Vacuum or N_2_		8–9 m	
		PET-SiOx/LDPE			11 m	
		PET/LDPE or PET-SiOx/LDPE	Oxygen absorber		≥ 12 m	
Cereals and cereal products	Muesli with chocolate and apricots	Paper bag: PAP + PP window	Air	20 °C, RH 55 %	2 m	[143]
		Pouch: PAP/Alu/PE			9 m	
		Can:PAP/Alu + LDPE lid				
	Fresh pasta	PS tray + PVC film	Air	8 °C	20 d	[120]
		PA/EVOH/LLDPE	CO_2_:N_2_ 22:78% MAP		40 d	
	Fresh pasta filled with cheese	Tray: EVOH/PS/PE wrapped in film: EVOH/OPET/PE	Air	4 °C	7–14 d	[108]
			CO_2_:N_2_ 50:50% MAP		42 d	
	Gluten-free fresh filled pasta	Tray: PETFilm: antifog PET film	Air	4 °C	14 d	[121]
		Tray: EVOH/PS/PEFilm: EVOH/OPET/PE	CO_2_:N_2_ 30:70% MAP		42 d	
Bakery wares	Sponge cake	PA/LLDPE	Combinations of oxygen scavengers with / without ethanol emitter	30 °C, RH 60%	≤42 d	[139]
		PVDC/PA/cPP				
	Sliced wheat bread	PET-SiOx/LDPE	Bread	20 °C	4 d	[130]
			Bread + preservatives		6 d	
			Ethanol emitter		24 d	
			Ethanol emitter + oxygen absorber		30 d	
	Ciabatta bread	OPA/PE	Air (control)	21 °C	5 d	[122]
			Air + ethanol spray		11 d	
			CO_2_:N_2_ 10:90% MAP		12 d	
			MAP + ethanol spray		13 d	
			Air + ethanol emitter		25 d	
			MAP + ethanol emitter		30 d	
	Wheat bread	HDPE/PE	-	25.8 °C, 275.5 lx, RH 31.2%	2 d	[144]
		Unpackaged bread	-		3 d	
		HDPE/Nanoparticles/PE	Ag-TiO_2_		>6 d	
	Calcium-enriched wholemeal bread	PA/PE bag + cardboard box	CO_2_:N_2_ 60:40% MAP	20 °C	24 d	[145]
	Whole wheat bread	PA/PE	N_2_	RT	2–3 w	[123]
	Part-baked flat bread (Sangak)	PA/PE	Air	25 °C	9 d	[124]
			CO_2_:N_2_ 20:80% MAP		18 d	
			CO_2_ 100% MAP		21 d	
	Sliced wheat bread	Tray: APET/EVOH/PEAntifog-film: PA/PE	Air without potassium sorbate & with 0.15% potassium sorbate	20 °C, RH 60%	14 d	[125]
			N_2_ 100% MAP, CO_2_:N_2_ 30:70% MAP, CO_2_:N_2_ 50:50% MAP, CO_2_:N_2_ 70:30% MAP, CO_2_ 100 %MAP;with & without potassium sorbate		21 d	
			Air with 0.30% potassium sorbate		>21 d	
	Bread	Plastic bag	E-Poly-L-Lysine Biofilms1.6/3.2/6.5 mg of E-Poly-L-Lysine /cm^2^	RT for 7 days inoculated with *A. parasitus*	+1 d	[131]
			E-Poly-L-Lysine Biofilms6.5 mg of E-Poly-L-Lysine /cm^2^	RT for 7 days inoculated with *P. expansum*	+3 d	
	Sliced wheat bread	PP/PET/LDPE	Star anise oil, thymol	25 °C inoculated with *P. roqueforti*	14 d	[132]
	Bread	Starch-based bionanocomposite film	Chitosan, grapefruit seed extract	25 °C, RH 59%	20 d	[133]
	Sliced white pan bread	PP bag	--	30°C	3 d	[134]
		PBAT-PLA bag				
			Trans-cinnamaldehyde		≥21 d	
	Bread	BOPP	--	25 °C, RH 75%	3 d	[135]
		PLA			6 d	
		PLA-PBSA bag	Thymol		7–9 d	
